# The influence of active and passive smoking on the cardiorespiratory fitness of adults

**DOI:** 10.1186/2049-6958-9-34

**Published:** 2014-06-09

**Authors:** Andresa Thier de Borba, Renan Trevisan Jost, Ricardo Gass, Fúlvio Borges Nedel, Dannuey Machado Cardoso, Hildegard Hedwig Pohl, Miriam Beatris Reckziegel, Valeriano Antonio Corbellini, Dulciane Nunes Paiva

**Affiliations:** 1 In Health Promotion, University of Santa Cruz do Sul (UNISC), Rio Grande do Sul, Brazil; 2 Physiotherapy, University of Santa Cruz do Sul (UNISC), Rio Grande do Sul, Brazil; 3 University of Santa Cruz do Sul (UNISC), Rio Grande do Sul, Brazil; 4 Dsc in Epidemiology, Federal University of Santa Catarina (UFSC), Santa Catarina, Brazil; 5 Physiotherapy, MSc in Medical Science. Assistant Professor, University of Santa Cruz do Sul (UNISC), Rio Grande do Sul, Brazil; 6 Professional Physical Education. DSc in Regional Development. Titular Professor, University of Santa Cruz do Sul (UNISC), Rio Grande do Sul, Brazil; 7 Professional Physical Education. MSc in Science of Human Movement, University of Santa Cruz do Sul (UNISC), Rio Grande do Sul, Brazil; 8 DSc in Chemistry, University of Santa Cruz do Sul (UNISC), Rio Grande do Sul, Brazil; 9 Physiotherapy, DSc Medical Science, University of Santa Cruz do Sul (UNISC), Rio Grande do Sul, Brazil

**Keywords:** Cardiovascular diseases, Exercise test, Oxygen consumption, Smoking

## Abstract

**Background:**

The aim of this study was to analyze the influence of active and passive smoking on cardiorespiratory responses in asymptomatic adults during a sub-maximal-exertion incremental test.

**Methods:**

The participants (n = 43) were divided into three different groups: active smokers (n = 14; aged 36.5 ± 8 years), passive smokers (n = 14; aged 34.6 ± 11.9 years) and non-smokers (n = 15; aged 30 ± 8.1 years). They all answered the Test for Nicotine Dependence and underwent anthropometric evaluation, spirometry and ergospirometry according to the Bruce Treadmill Protocol.

**Results:**

VO_2max_ differed statistically between active and non-smokers groups (p < 0.001) and between non-smokers and passive group (p=0.022). However, there was no difference between the passive and active smokers groups (p=0.053). Negative and significant correlations occurred between VO_2max_ and age (r = - 0.401, p = 0.044), percentage of body fat (r = - 0.429, p = 0.011), and waist circumference (WC) (r = - 0.382, p = 0.025).

**Conclusion:**

VO_2max_ was significantly higher in non-smokers compared to active smokers and passive smokers. However, the VO_2_max of passive smokers did not differ from active smokers.

## Background

Cardiorespiratory impairment increases morbidity and is an independent predictor of all-cause mortality [1]. Individuals with low cardiorespiratory capacity are more likely to develop systemic arterial hypertension (SAH) [2], diabetes [3], and metabolic syndrome [4] and have high mortality rates due to cardiovascular disease [5] and cancer (CA) [6] than individuals with high cardiorespiratory capacity. One way to evaluate cardiorespiratory ability is through the cardiopulmonary exercise test (CPET) or ergospirometry. Its effectiveness lies in its reflection of the strength of the cardiorespiratory system and its changes during exercise. One of the parameters used to estimate cardiopulmonary functional capacity is the maximum consumption of oxygen (VO_2max_) [7,8]. VO_2max_ reflects the maximum capacity of absorption, transportation and consumption of oxygen (O_2_). VO_2max_ is the most influential parameter of a person’s physical conditioning and is an independent and objective measure for the prognosis of cardiovascular disease [8,9].

Smoking affects the response to the CPET [10,11]. Smokers have lower aerobic capacity and, thus, less oxygen supply while they are exercising. Smoking also requires an additional energetic cost, which is caused by greater respiratory muscle work. Quitting smoking and practicing physical activities regularly can make aerobic function return to normal values [12]. The effects of passive smoking on cardiorespiratory capacity, however, are rarely addressed. A recent randomized clinical trial identified cardiorespiratory and immunological changes in healthy non-smoking individuals; these changes appeared immediately after they were exposed to cigarette smoke [13]. In 2008, Ren et al. [14] performed one of the first studies to evaluate the hemodynamics and cardiopulmonary function of flight attendants who were exposed to cigarette smoke for over five years. These authors noted a connection between passive smoking and SAH, but they did not show significant cardiopulmonary impairment.

Therefore, the main goal of this study was to analyze the influences that active and passive smoking have on cardiorespiratory responses in asymptomatic adults throughout a sub-maximal-exertion incremental test on a treadmill.

## Methods

This transversal study was composed of smokers, non-smokers and passive smokers, aged between 18 and 50 years and of both genders. In this study, among participants active smokers were considered those who had smoked at least five cigarettes/day for 10 years prior to the study (active smoker group); whereas passive smokers were those who lived with at least one smoker or interacted with a smoker at work for at least three years prior to the study (passive smoker group); and the non-smokers were those who had never smoked (non-smoker group). Individuals who had cardiorespiratory disease, trauma-orthopedic dysfunction, diabetes or CA were excluded from the study, as were ex-smokers and individuals who had been exposed to toxic inhalants.

The study enrolled 43 adults from the target population. To calculate the sample size, we evaluated a difference in the average cardiorespiratory function in six subjects, considering a standard deviation of 5%, a statistical power of 90% and confidence interval of 95% [15]. This study was approved by the Research Ethics Committee of the University of Santa Cruz do Sul, and free informed consent was obtained under protocol number 2682/2010 from every participant.

Initially, the level of nicotine dependence was evaluated using the Fagerström Test for Nicotine Dependence (FTND) [16-19]. The number of years the individuals smoked and, in case of passive smoking, the period they were exposed to cigarette smoke (minutes/day and the number of years) were also recorded [20,21]. All exams were performed by qualified professionals.

### Anthropometric evaluation

The anthropometric evaluation was performed by measuring body mass (kg) and height (cm) using an anthropometric scale. Body mass index (BMI) was calculated following the criteria established by the World Health Organization [22]. Using a non-elastic measuring tape, waist circumference (WC) was measured bilaterally at the midpoint between the iliac crest and the lower costal margin, according to the criteria proposed by Heyward [23], and was classified according to the 1^st^ Brazilian Guideline for Metabolic Syndrome Diagnosis and Treatment [24].

To determine the body fat percentage (%F), seven skin folds were measured. These measures were different for men and women, according to Jackson and Pollock’s protocol [25,26], and were performed with the Lange® Caliper (Multimed, Skinfold Caliper, Gays Mills, WI, USA). Measures were taken three times in a rotational sequence to obtain an average for each individual at each location. Jackson and Pollock’s approach [25] was used to calculate body density (BD), and the Siri equation was used to obtain %F.

### Lung function evaluation

Lung function was evaluated using a portable spirometer (EasyOne®, Model 2001 Diagnostic Spirometer, NDD Medical Technologies, Andover, MA, USA) following the protocol of the American Thoracic Society [27]. The following variables were evaluated: forced vital capacity (FVC), forced expiratory volume (VEF_1_), VEF_1_/FVC ratio and peak expiratory flow (PEF). The curves for these parameters were compared to reference values for this population [28].

### Cardiorespiratory capacity

Treadmill cardiopulmonary exercise testing was performed using the Bruce protocol [9] and was used to identify and classify cardiorespiratory capacity (Ecafix® EG treadmill-700X, São Paulo, SP, Brazil). An aneroid sphygmomanometer was used to measure systolic arterial pressure (SAP) and diastolic arterial pressure (DAP) every 3 minutes throughout the test [29] and respiratory gas analysis was recorded continuously throughout the test. Simultaneous, respiratory gas analysis was performed using breath by breath analysis of O_2_ and CO_2_ on a TEEM 100 Metabolic Analysis System® instrument (Aero Sport, Ann Arbor, MI, USA), as previously validated by Novitsky [30]. All exams were performed in the morning at a controlled room temperature. The oxygen and carbon dioxide sensors were calibrated before each exercise test [31]. The VO_2max_ was defined as the maximum O_2_ consumption reached during the last minutes of exercise during the exertion test. VO_2max_ was estimated, and the participants were classified according to their functional capacity, following criteria established by Pollock and Wilmore [32]. VO_2max_ was normalized to age, height and gender using the formula published by Jones et al. [33]. The highest HR reached during the exercises was defined as HR reached (HRreached). The highest RQ was termed RQ peak (RQpeak). The test was interrupted if the subject indicated any discomfort that could prevent him/her from continuing the test or when the individual reached 85% of the maximum HR set by the Karvonen formula (220–age). All individuals were informed and educated about the procedure of the test.

### Level of physical activity

To quantify their physical activity, all volunteers answered the International Physical Activity Questionnaire (IPAQ–short version). The short version of the IPAQ addresses the number of days and minutes spent practicing physical activities as recreational and occupational activities, transportation and house hold duties. The score was obtained by summing the number of days and minutes or hours of physical activities performed during the week prior to the completion of the questionnaire. The levels of physical activity were classified as sedentary, insufficiently active, active, and intensely active by taking into consideration the frequency, intensity, and length of these activities [34].

In addition to the IPAQ and the Test for Nicotine Dependence, the participants were asked to provide personal details such as age, gender, race and educational background.

### Statistical analysis

The statistical software SPSS (version 18.0, SPSS Inc., Chicago, Illinois, USA) was used for statistical analysis. The Shapiro-Wilk test was used to determine the normality of the data distributions. Descriptive statistics (mean, standard deviation and absolute frequency) were calculated for most parameters. Fisher’s exact test was used to compare the categorical variables among groups. One-way analysis of variance (ANOVA) was used to compare mean VO_2max_, followed by Tukey’s *post-hoc* test and student’s T-test when necessary. Pearson’s correlation test and Analysis of Covariance (ANCOVA) was also performed to compare VO_2max_ and its covariates age, %F, WC and level of physical activity, among groups. P < 0.05 was considered significant.

## Results and discussion

The sample was composed of 43 Caucasian individuals with an average age of 33.5 ± 9.6 years. The individuals were allocated evenly into the active smoker group (n = 14; aged 37.2 ± 7.7 years), passive smoker group (n = 14; aged 33.1 ± 11.8 years) and non-smoker group (n = 15; aged 30.4 ± 8.2 years). Table 1 shows their demographic, anthropometric and basic cardiopulmonary characteristics. Most individuals from the sample (83.3%) had WC within the normal range, but the non-smoker group and the active smoker group had significantly different WC (p = 0.04). The %F was classified as excellent, good, above average or average in 37 individuals (72.9%). The spirometric analysis revealed values within the normal range in all individuals.

**Table 1:** Basic characteristics of the study sample

**Characteristics**	**Non-smokers**	**Passive smokers**	**Active smokers**	**p**
	**(n = 15)**	**(n = 14)**	**(n = 14)**	
Male gender, n (%)	6 (40)	4 (28.5)	4 (28.5)	0.97
Age (years old)	30,4 ± 8.2	33.1 ± 11.8	37.2 ± 7.7	0.16
BMI (kg/m^2^)	23.6 ± 4.6	24.3 ± 4.2	27.2 ± 3.9	0.06
WC (cm)	74.1 ± 12.1^†^	77.2 ± 10.7	84.1 ± 8.5^†^	0.04
%F	20.9 ± 8.4	23.1 ± 6.6	24.1 ± 6.2	0.51
SAP resting (mmHg)	117.9 ± 10.4	120.3 ± 12.6	120.9 ± 9.3	0.73
DAP resting (mmHg)	76.1 ± 9.4	74.6 ± 7.4	74.6 ± 9.1	0.88
FVC (% pred)	95.8 ± 21.1	105 ± 15.8	104.2 ± 13.2	0.28
FEV_1_ (% pred)	89.4 ± 19.5*	102.8 ± 15.4	103 ± 10.8*	0.03
FEV_1_/FVC (% pred)	92.6 ± 9.2	97.4 ± 6.5	97.1 ± 5.4	0.15

BMI, body mass index; DAP, diastolic arterial pressure; FEV_1_, forced expiratory volume in the first second; FEV_1_/FVC, ratio between FEV_1_ and FVC; FVC, forced vital capacity; %F, percentage of body fat; SAP, systolic arterial pressure; WC, waist circumference. Values are expressed as the mean ± SD. ^†^Significant difference between non-smokers and active smokers (p = 0.04). *Significant difference between non-smokers and active smokers (p = 0.03). Significance was accepted at p < 0.05.

According to the FTND, nine out of the 16 active smokers had a medium/high/extremely high degree of nicotine addiction. Instead, in seven individuals, the addiction degree was low/awfully low. All active smokers had been smoking for over 10 years. The average number of cigarettes smoked was 20.6 ± 9.6 cigarette/day002E.

Among the passive smokers, most individuals were exposed to cigarette smoke for over an hour per day (62.5%). All of them had experienced this exposure for over three years. Damage caused by smoking affects not only active smokers but also non-smokers who are exposed to cigarette smoke at home, at work, in leisure environments, at school and in other enclosed public areas [35]. Passive smoking has been considered a significant risk factor for the development and evolution of cardiopulmonary dysfunctions, including loss of endothelial function and coronary artery disease [36,37].

There is a connection between passive smoking and loss of lung function [38,21-41]. However, there is little information about the impact of passive smoking on cardiorespiratory capacity. Furthermore, studies that have quantified the exposure to cigarette smoke have used different methods; these differences hamper and complicate the interpretation of the data.

Flouris et al. [13] evaluated cardiorespiratory and immunological responses during physical activity training after cigarette smoke exposure. Seventeen individuals of both genders were exposed to cigarette smoke in a controlled environment with a concentration of carbon monoxide similar to what is found in restaurants and bars. These individuals were monitored during and after the completion of moderate physical activities. The authors found cardiorespiratory (increased RQ) and immunological (increased interleukins) changes immediately after exposure to cigarette smoke.

In contrast, some studies have been inconsistent when showing significant cardiopulmonary effects caused by exposure to cigarette smoke. One of the first studies to evaluate cardiopulmonary hemodynamics and cardiopulmonary capacity related to residual effects of passive smoking, Ren et al. [14] studied 79 flight attendants exposed to cigarette smoke inside aircraft cabins for over five years reported that passive smoking was linked to SAH but did not cause hemodynamic, pulmonary or systemic consequences. However, in our study, there was evidence of significant differences in CPET results between passive smokers and non-smokers, as well as between non-smokers and active smokers groups.

**Figure 1:**
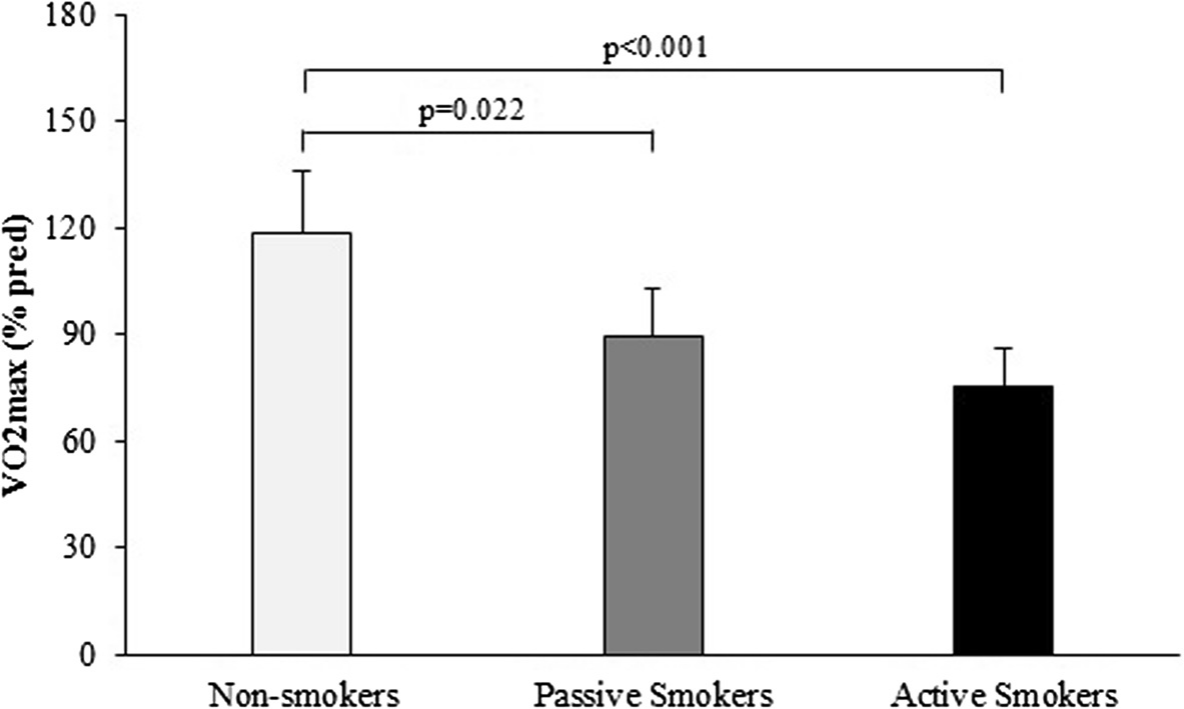
**Comparison of VO2**_**max **_**among groups after adjustment for body mass index and waist circumference.**

Table 2 shows the values obtained during the CPET in each group. When evaluating VO_2max_ in active, passive and non-smoking individuals using CPET, significant differences were observed between groups (p < 0.001) (Figure 1). When comparing VO_2max_ between groups, after adjustment for age, %F, WC and level of physical activity showed a significant difference between the non-smoker and passive smoker group (p = 0.022) even as non-smoker and active group (p < 0.001). The passive and active smoker groups showed a not significantly VO_2max_difference (p = 0.053).

**Table 2:** Variables obtained during the cardiopulmonary exercise test

**Characteristics**	**Non-smokers**	**Passive smokers**	**Active smokers**	**p**
	**(n = 15)**	**(n = 14)**	**(n = 14)**	
VO_2max_ (% pred)*	118.44 ± 38.11^†^**	89.75 ± 7.4^†^	75.13 ± 17.78**	<0.001
RQ peak (l·min^-1^)	1.13 ± 0.3	1.2 ± 0.4	1.1 ± 0.3	0.82
HR resting (bpm)	70.6 ± 14.9	73.6 ± 11.8	77.6 ± 13.8	0.38
HR reached (bpm)	159.1 ± 19.6	160.1 ± 13.6	152.7 ± 13.9	0.42
SAP resting (mmHg)	117.9 ± 10.4	120.3 ± 12.6	120.9 ± 9.3	0.73
SAP reached (mmHg)	151.3 ± 11.2	148.9 ± 18.4	147.1 ± 9.1	0.70
DAD resting (mmHg)	76 ± 9.4	74.6 ± 7.4	74.6 ± 9.1	0.88
DAP reached (mmHg)	84.3 ± 5.6	85.3 ± 6.3	84.6 ± 7.4	0.91

DAP, diastolic blood pressure; HR; heart rate; RQ, respiratory quotient; SAP, systolic arterial pressure; VO_2max_, maximum oxygen consumption;. Values are expressed as mean ± SD. p, p-value obtained through analysis of variance (ANOVA). *Analysis of covariance (ANCOVA) was also performed to compare VO_2max_ and its covariates age, %F, WC and level of physical activity, among groups. Significance was accepted at p < 0.05. ^†^Significant difference between non-smokers and passive smokers (p = 0.022). **Significant difference between non-smokers and active smokers (p < 0.001).

Several studies have reported a correlation between VO_2max_ and smoking. Kobayashi et al. [15] evaluated the impact of smoking on cardiorespiratory responses during sub-maximal exercise activities. In their study, 18 healthy men were evaluated (nine smokers: average of 15 cigarettes/day for over five years; nine non-smokers). They found that smoke harmed cardiorespiratory function while exercising due to a reduction of the body capacity to carry O_2_. Laukkanen et al. [6] evaluated determinants of cardiorespiratory capacity in men aged 42 to 60 years and found an inverse correlation between smoking and VO_2max_[42]. Louie [43] conducted a running test in 27 teenagers smokers or non-smokers and showed that, even in young individuals, smoking was associated with a significant reduction of cardiopulmonary activity and exercise tolerance; this effect remained even at light smoking levels.

According to Kobayashi et al. [15], smoking significantly worsens cardiorespiratory function during moderate to severe exercises. This impairment happens because the body’s capacity to carry O_2_ is reduced, resulting in a higher anaerobic metabolism, which may lead to injuries on the inside walls of blood vessels, making them more rigid [44]. In our study, there was no difference in RQ among groups, although it was slightly higher in active smokers.

Unverdorben et al. [45] observed higher HR and resting SAP in active smokers than in non-smokers. This fact can be explained by the action of nicotine, which activates the sympathetic nervous system, leading to the release of epinephrine and norepinephrine. The increase in HR may cause heart diseases, which are associated with smoking [46,47]. However, the increase in resting HR may be influenced by psychological stress before performing the CPET. In this study, there were no significant differences among groups in resting HR or resting SAP, although the active smoker group had slightly higher averages.

In our study, the maximum HR reached during the exertion test of the active smoker group was lower compared with the others, as observed by Unverdorben et al. [45]. Smokers who suffer from chronotropic incompetence have a significantly increased risk of death and coronary disease, and HR is an important predictor of all-cause mortality [46]. A longitudinal study performed in 2003 indicated that smoking was negatively associated with the maximum HR obtained during CPET in men and women between 13 and 36 years and that smokers reached a maximum HR lower than in non-smokers [11]. According to Srivastava et al. [46] and Lauer et al. [47], smoking also modifies the chronotropic response to exercise.

Similar to maximum HR, the maximum SAP reached was slightly but not significantly lower in the active and passive smoker groups. This finding indicates that smokers have difficulty in maintaining appropriate cardiac output, which can be explained by chronotropic incompetence. Furthermore, the maximum DAP reached was higher in the active and passive smoker groups compared with the non-smokers, possibly due to a greater vasoconstrictor tone.

**Figure 2:**
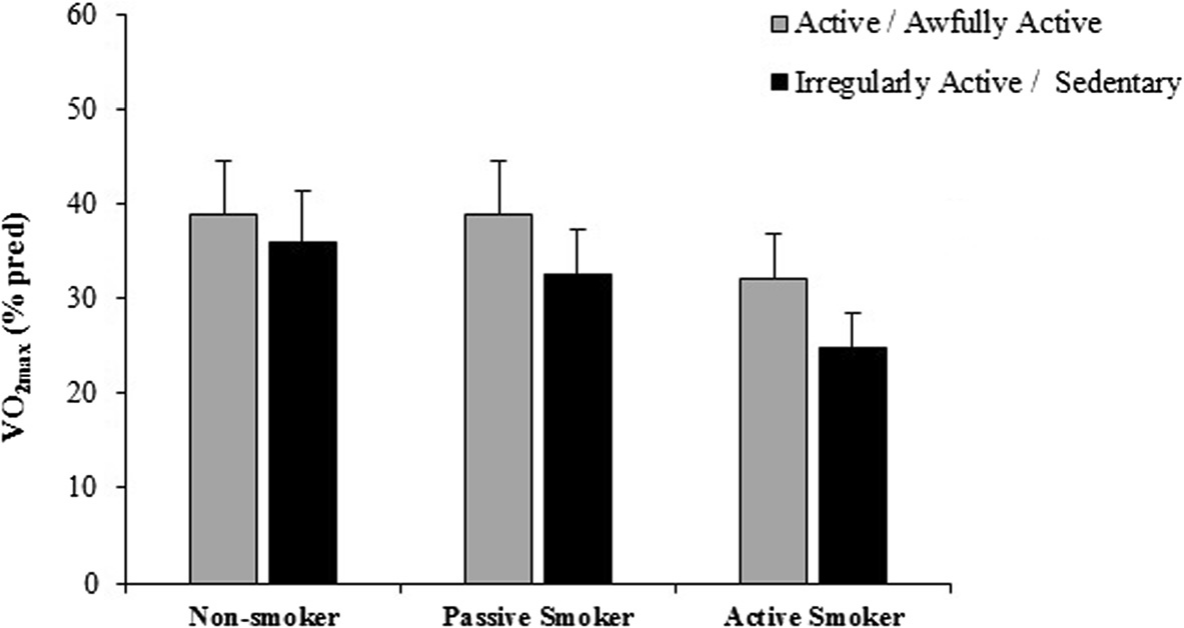
**Comparison of VO2**_**max **_**(% predicted) stratified by level of physical activity in each group analysed.**

According to the IPAQ classifications, most individuals were classified as sedentary or irregularly active (68.8%), with a smaller proportion of active and awfully active individuals (31.3%). No significant results were found when comparing VO_2max_ stratified by level of physical activity between any of two groups (p = 0.063) (Figure 2).

In our study, we expected to find an association between smoking and sedentarism because smoking is described as more prevalent in sedentary individuals and because physical activities can be a protective factor against beginning habits such as smoking [20]. The lack of association between smoking and sedentariness may have been related to the low degree of nicotine dependence because seven out of 15 active smokers had a low or awfully low degree of nicotine dependence. The IPAQ instrument used to identify the level of physical activity takes into consideration several types of activities, including occupational and transportation activities, which represent a large proportion of the total activities that inhabitants of developing countries engage in [48]. This description could explain the number of active/awfully active individuals in this study.

As seen in Table 3, in the whole sample, VO_2max_ was correlated with age, %F and WC respectively. According to Lee et al. [49], cardiorespiratory capacity depends on modifiable factors (e.g., physical activity, smoking, obesity and health conditions) and non-modifiable factors (e.g., age, gender and genotype). After a person’s maximum cardiorespiratory capacity has been reached between 20 and 30 years, a decline associated with age begins, especially if there are body weight gain and reduction of physical activity [50]. This study confirmed that greater age and %F were associated with lower VO_2max_. Although there was an association among age, %F and WC, these variables did not affect the outcome of VO_2max_ in the groups. Furthermore, no significant differences were observed between the level of physical activities and VO_2max_, even though VO_2max_ was lower in sedentary individuals than in active ones. These findings support previous studies [51,52]. Cheng et al. [51] reported that smokers and sedentary individuals had the worst results during the Maximum Exertion Test and spirometry.

**Table 3:** **Correlations between VO**_**2max**_** and age, percentage of body fat and waist circumference**

**VO**_**2max**_** (% pred)**	**r**	**p**
Age (years)	-0.401	0.044*
%F	-0.429	0.011*
WC (cm)	-0.382	0.025*

VO_2max_, maximum oxygen consumption; r, Pearson’s correlation coefficient. Significance was accepted at p < 0.05.

*Analysis of Pearson’s correlation test and Analysis of Covariance (ANCOVA) was also performed to compare VO_2max_ and age, percentage of body fat and waist circumference, among groups.

It is important to highlight the methodological limitations of this study. A cross-sectional study is fast and less expensive than other studies, but it is limited by its brief time frame. For this reason, it was not possible to evaluate the temporal relationships between causes and effects. Regarding the determination of tobacco intake of the subjects in this study it was not possible to quantify the level of urinary cotinine, and therefore we used the Fagerström Test for Nicotine Dependence, which is a validated tool to quantify tobacco load [53,54]. The use of questionnaires also limits the strength of our data because questions might not be answered completely due to a lack of understanding or recall by the respondents caused by their low education level or inability to remember some aspects of the evaluation or even because they unconsciously overestimated or underestimated their activities.

Recall bias related to active and passive smoking must also be considered. The ability to determine one’s exposure to tobacco smoke is potentially subject to information bias, which may limit the interpretation of the results. However, we classified passive smokers according to the participants’ self-reports, as in other studies [20,21]. In addition to costing less than other methods, questionnaires to verify passive smoking are valid instruments for projecting the level of cigarette smoke exposure [36,55,56].

In our study the significant difference between passive smokers and non-smokers could be explained by many factors like the amounts of chemical substances coming from environmental smoke that depend on number of smokers, level of cigarette consumption, types of cigarettes smoked (with or without strainer, tar content or nicotine content), proximity to the passive smoker, duration of exposure, size of the exposure space, characteristics of the ventilation system, age of the person exposed, frequency of air exchange in the closed environment, and use of air purifiers [57,58,36]. These variables were not measured in our study, which may have limited the generalizability of our results.

## Conclusions

This study, which aimed to identify the effects of smoking on the cardiorespiratory capacity of active and passive smokers, found significant difference between active smokers and non-smokers. Nevertheless, we found no difference between active and passive smokers. VO_2max_ was negatively correlated with age, %F and WC. In contrast, there was no significant correlation between the level of physical activity and VO_2max_, although sedentary people showed lower maximum oxygen consumption than the more active ones. Because the literature on passive smokers is limited, more studies on the effects of passive smoking on cardiopulmonary fitness are necessary and justified.

## Competing interests

The authors declare that they have no competing interests.

## Author’s contributions

ATB, FBN, HHP, MBR, VAC and DNP contributed to design the study, write the manuscript and revise editing. ATB, RTJ, RG and DMC contributed to the collection and analysis of statistical data. ATB, RTJ, RG, DMC and DNP contributed to the interpretation of data and revision of the manuscript. ATB, RG, FBN, DMC, HHP, MBR, VAC and DNP performed the final revision of the manuscript. All authors read and approved the manuscript.
